# Baicalein induces CD4^+^Foxp3^+^ T cells and enhances intestinal barrier function in a mouse model of food allergy

**DOI:** 10.1038/srep32225

**Published:** 2016-08-26

**Authors:** Min-Jung Bae, Hee Soon Shin, Hye-Jeong See, Sun Young Jung, Da-Ae Kwon, Dong-Hwa Shon

**Affiliations:** 1 Korea Food Research Institute, 1201-62, Anyangpangyo-ro, Bundang-gu, Seongnam-si, Kyeonggi-do 463-746, Korea; 2 Institutes of Entrepreneurial BioConvergence, School of Biological Sciences, Seoul National University, Seoul 151-742, Korea; 3 Food Biotechnology Program, Korea University of Science and Technology, Daejeon 305-350, Korea

## Abstract

The incidence of food allergy, which is triggered by allergen permeation of the gastrointestinal tract followed by a T-helper (Th) 2-mediated immune response, has been increasing annually worldwide. We examined the effects of baicalein (5,6,7-trihydroxyflavone), a flavonoid from *Scutellaria baicalensis* used in oriental herbal medicine, on regulatory T (Treg) cell induction and intestinal barrier function through the regulation of tight junctions in a mouse model of food allergy. An allergic response was induced by oral challenge with ovalbumin, and the incidence of allergic symptoms and T cell-related activity in the mesenteric lymph nodes were analyzed with and without the presence of baicalein. Our results demonstrated that the administration of baicalein ameliorated the symptoms of food allergy and attenuated serum IgE and effector T cells. However, Treg-related factors were up-regulated by baicalein. Furthermore, baicalein was shown to enhance intestinal barrier function through the regulation of tight junctions. We also found that baicalein treatment induced the differentiation of Treg cells via aryl hydrocarbon receptors (AhRs). Thus, the action of baicalein as an agonist of AhR can induce Treg differentiation and enhance barrier function, suggesting that baicalein might serve as an effective immune regulator derived from foods for the treatment of food allergy.

Baicalein is a natural flavone (a type of flavonoid) isolated from *Scutellaria baicalensis*. It is also known as a dietary flavonoid and phytonutrient along with quercetin, apigenin, luteolin, and myricetin as it is found in high concentrations in various foods such as vegetables, fruits, beverages, and many traditional herbs^1^,^2^. Baicalein has been found to have multiple beneficial properties including antioxidant^3^, anti-inflammatory^4^, anti-allergic^5^, and anti-cancer activities^6^. In particular, the anti-allergic effects of baicalein have been verified in asthma, atopic dermatitis, and both IgE-mediated and systemic allergic responses^7^,^8^,^9^. However, the molecular and cellular mechanisms of the protective effects of baicalein on the food allergy have not yet been clearly elucidated.

The incidence of food allergy is increasing annually and has been estimated to affect approximately 8% of children and 2% of the adult population^10^,^11^. The allergic response to food is triggered by allergen permeation into the intestinal epithelium via paracellular diffusion. The permeated allergens are presented to CD4^+^ T cells by antigen presenting cells; the CD4^+^ T cells then differentiate into T-helper 2 (Th2) cells, which produce IL-4, IL-5, and IL-13. These Th2 cytokines induce B-cell production of antigen-specific IgE antibodies, which bind to their high-affinity receptor FcεRI on mast cells and cross-link with the allergens. Such allergen provocation triggers mast cell degranulation, releasing histamine, prostaglandin, and leukotriene release, which causes food allergy symptoms^12^. Anti-histamine drugs have been widely used to treat these allergic responses. However, as these treatments are often associated with multiple side-effects and are not universally efficacious, the initial steps of allergic responses such as the inhibition of Th2 immune responses by regulatory T (Treg) cells or suppression of allergen penetration by tight junctions (TJs) might represent novel target mechanisms for the prevention of allergic diseases as an alternate strategy in patient care.

Treg cells, which strongly express the transcription factor forkhead box protein P3 (Foxp3)^13^,^14^, are crucial to maintaining immune tolerance, which is associated with the alleviation of food allergy, oral tolerance, and autoimmune suppression^15^. Disruption or dysfunction of Treg cells often leads to Th1-, Th2-, and Th17-mediated disorders such as rheumatoid arthritis^16^, allergic inflammation^17^, inflammatory bowel disease (IBD)^18^, and type 1 diabetes^19^. Therefore, the induction of CD4^+^Foxp3^+^ Treg cells represents a critical target for the regulation of immune response. Notably, probiotics^20^, dietary factors^21^, and plant-derived compounds^22^ regulate the immune response by inducing Foxp3 to stimulate the differentiation and functionality of Treg cells. These findings might support the application of various immune-modulation tactics in the treatment of immune diseases, especially in food allergy. Furthermore, the increase of intestinal permeability and the destruction of intestinal barrier function have been known as risk factors for the development of food allergy^23^,^24^. Thus, the enhancement of intestinal barrier function might also attenuate or ameliorate various allergic disorders including food allergy by suppressing allergen permeation^25^.

In this study, we examined the anti-allergic effects of baicalein in a mouse model of food allergy, on the differentiation of naïve CD4^+^ T cells to Treg cells via Foxp3 induction, and on intestinal barrier function via the regulation of TJs. Our results suggest that the two targets, Treg cells and TJs, might be useful in regulating food allergic responses.

## Results

### Baicalein reduces OVA-induced symptoms in a mouse model of food allergy

**Figure 1:**
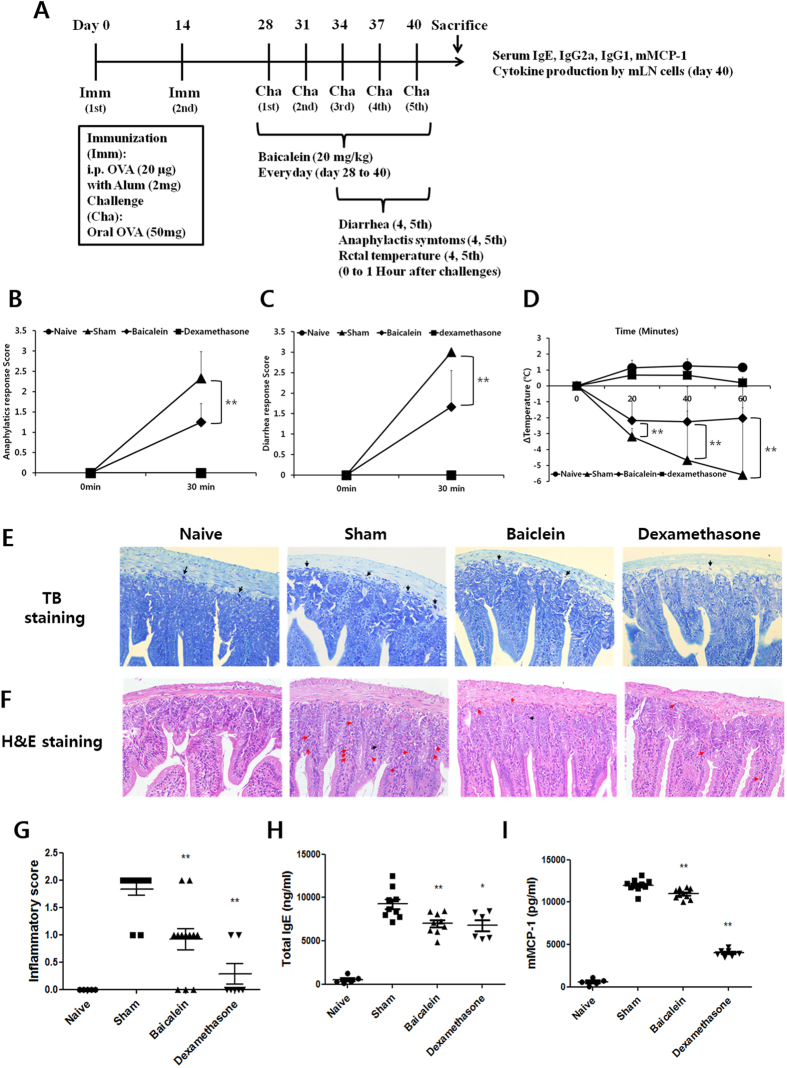
Baicalein reduced OVA-induced symptoms in a mouse model of food allergy. (**A**) Symptoms of food allergy were induced in BALB/c mice by immunization (twice with OVA and Alum by i.p. injection) and challenges (five times with *per os* (p.o.) OVA). Anaphylactic response (**B**), diarrhea (**C**), and rectal temperature (**D**) were evaluated for 60 min by at least three investigators and scored. The jejunum tissues were isolated and stained with TB and H&E. (**E**) TB staining shows mast cells (black arrows) migrating to the lamina propria. (**F**) H&E staining indicates neutrophil (red arrows) and eosinophil (black arrows) infiltration of the jejunum. (**G**) Inflammation was scored. To analyze the allergic mediators in the serum, IgE (**H**) and mMCP-1 (**I**) were measured by ELISA. Each value represents the means ± SD (*n* = 5–12). Bars indicate significant differences from the control at **p < 0.01.

OVA-induced food allergy symptoms were evaluated and scored for diarrhea, anaphylactic response, and rectal temperature on the 5^th^ challenge for 60 min. Severe symptoms of OVA-induced food allergy were observed in the sham group (diarrhea, 3 points; anaphylactic response, 2.3 ± 0.65 points). In contrast, the baicalein-treated group showed significant suppression of the diarrhea (1.6 ± 0.88 points) and anaphylactic response (1.25 ± 0.45 points) (Fig. 1B,C). In addition, the rectal temperature in the sham group decreased by −6.75 °C compared to the naive group, whereas in the baicalein-treated group, the change in rectal temperature was −3.18 °C (Fig. 1D). We also observed histopathological changes in the jejunum, ileum, and colon by TB staining corresponding to mast cell migration. The number of migrating mast cells increased in the jejunum (especially in the lamina propria), but not in the ileum and colon, of the sham group (data not shown). This effect was reduced by baicalein treatment (Fig. 1E). H&E staining illustrates histological changes such as mucosal and crypt damage and lymphocyte infiltration. Here, the sham group showed marked inflammation and lymphocyte infiltration in comparison to the naive group (Fig. 1F). Baicalein attenuated the inflammatory response in the jejunum, and reduced the inflammation scores therein from 1.83 (sham group) to 0.91 (Fig. 1G). The anti-allergic effects of baicalein were also indicated by the decreased levels of serum IgE and mMCP-1 in treated mice (Fig. 1H,I).

### Baicalein-mediates immunosuppression in the mLNs of OVA-induced mice

**Figure 2:**
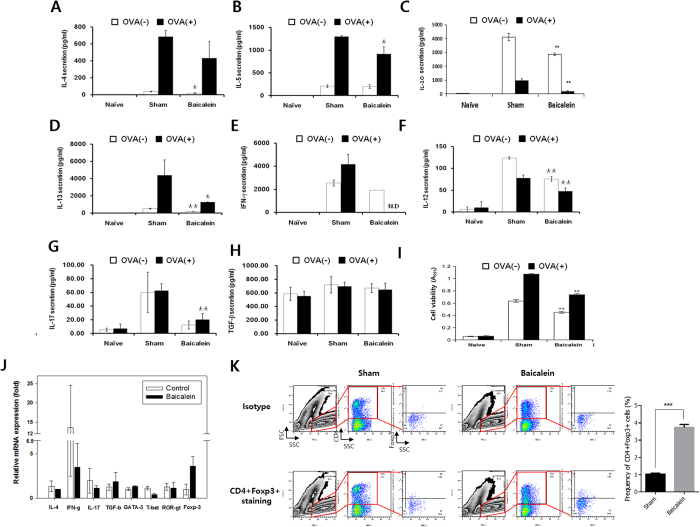
Baicalein-mediated immunosuppression in the mLNs of OVA-induced mice. Immune cells isolated from the mLN were cultured in the presence of OVA for 72 h. Cytokine levels of IL-4 (**A**), IL-5 (**B**), IL-10 (**C**), IL-13 (**D**), IFN-γ (**E**), IL-12 (**F**), IL-17 (**G**), and TGF-β (**H**) were measured by ELISA, and the cell viability (**I**) was evaluated by MTT assay. (**J**) The mRNA levels of cytokines and transcriptional factors were analyzed by real-time RT-PCR. (**K**) Lymphocytes isolated from mLNs were stained for CD4 and Foxp3 and analyzed by flow cytometry. Each value represents the means ± SD (*n* = 3). Bars indicate significant difference from the control at *p < 0.05 and **p < 0.01.

We next investigated the cytokine patterns in mLNs from mice with OVA-induced food allergy. All Th2-related cytokines (IL-4, IL-5, IL-10, and IL-13) were increased by the allergy induction and inhibited by baicalein (Fig. 2A–D). Th1- (IFN-γ and IL-12) and Th17-related cytokines (IL-17) also increased in the sham group and decreased with baicalein (Fig. 2E–G). However, TGF-β, which is produced by Treg cells, was increased by baicalein in comparison to the levels observed in the sham group (Fig. 2H,I). Furthermore, the transcript levels of Th1-, Th2-, and Th17-related cytokines and transcriptional factors were reduced by baicalein (Fig. 2J), whereas the Treg-related cytokine TGF-β and transcription factor Foxp3 were increased. Baicalein increased the population of CD4^+^Foxp3^+^ T cells to 6.35% compared with that in the sham group (2.58%) (Fig. 2K). These results demonstrated that oral administration of baicalein may suppress T cells including Th2 cells by inducing their differentiation from naive CD4^+^ T cells to CD4^+^Foxp3^+^ Treg cells, thus attenuating food allergy symptoms.

### Baicalein enhances intestinal barrier function in OVA-induced mice

Intestinal barrier function is very important to maintain homeostasis; the destruction of the barrier function leads to intestinal disorders such as leaky gut syndrome, IBD, and food allergy. However, although the change of intestinal permeability by the destruction of barrier function has been considered to be a risk factor for the development of food allergy, it has not yet been substantiated with experimental data^23^,^24^. We therefore investigated whether baicalein could regulate intestinal barrier function in our mouse model of food allergy by examining the TJ proteins claudin-1, occludin, ZO-1, and JAM-1 in the intestinal epithelium. As predicted, we found that their expression was reduced in OVA-induced mice, whereas administration of baicalein restored expression of claudin-1, ZO-1, and JAM-1 (Table 1). In addition, occludin mRNA expression was strongly enhanced by baicalein treatment to 3.34 and 5.54 fold compared with that of the naive and sham groups, respectively. These results represent the first scientific evidence that enhancement of TJ proteins might contribute to attenuate food allergy. We suggest that baicalein might be able to inhibit the permeation allergens such as OVA in food allergy through enhancing TJ protein levels.

### Baicalein induces CD4^+^Foxp3^+^ T cell differentiation

**Figure 3:**
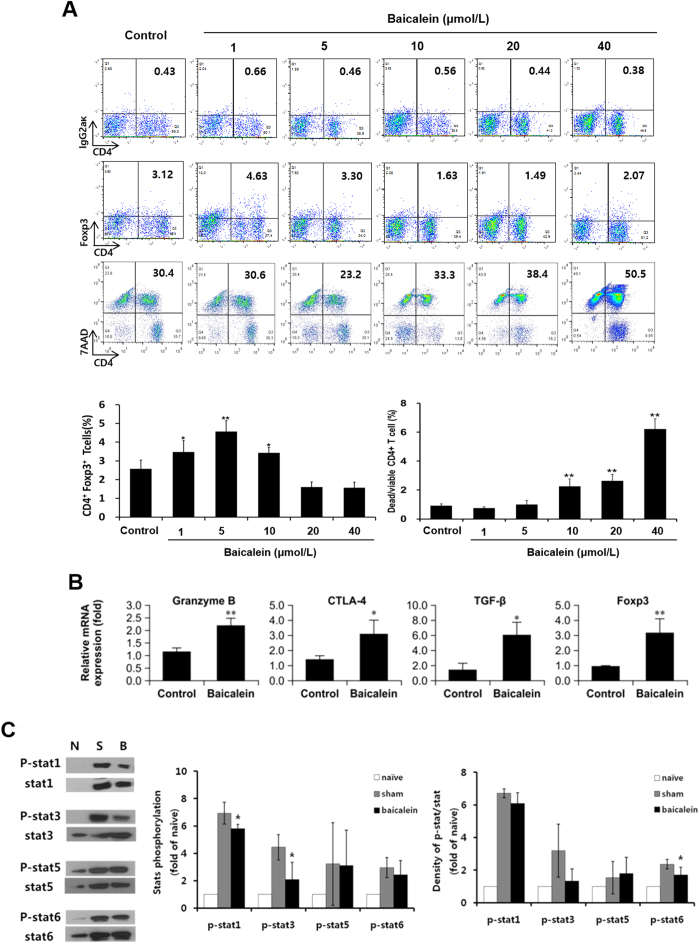
Baicalein induced CD4^+^Foxp3^+^ T cell differentiation. Naive CD4^+^ T cells (CD4^+^CD62L^+^ T cells) from the spleen and mLN were treated with 0–40 μmol/L baicalein, 10 μg/mL plate-bound anti-CD3 mAb, and 2 μg/mL soluble anti-CD28 mAb for 72 h, then analyzed for CD4 and Foxp3 and CD4 and 7AAD expression by flow cytometry (**A**). Naive CD4^+^ T cells were treated with 5 μmol/L baicalein, 10 μg/mL anti-CD3 mAb, and 2 μg/mL anti-CD28 mAb for 48 h. (**B**) Treg-specific GranB, CTLA-4, TGF-β, and Foxp3 were evaluated. (**C**) Phosphorylated and total STAT1, STAT3, STAT5, and STAT6 were detected by western blotting. This experiment was performed independently at least three times. The STATs were quantified as band density analyzed with ImageJ software. Each value represents the means ± SD (*n* = 3). Bars indicate significant difference from the control at *p < 0.05 and **p < 0.01.

We next investigated Treg induction by 0–40 μmol/L baicalein and found that 1–10 μmol/L baicalein dramatically increased the population of CD4^+^Foxp3^+^ T cells, although 10–40 μmol/L baicalein produced only weak induction (Fig. 3A). Thus, we used 7-ADD staining and 3-(4,5-dimethylthiazol-2-yl)-2,5-diphenyltetrazolium bromide (MTT) assays to investigate whether baicalein causes Treg cell death. We found that baicalein induced cell death in CD4^+^ T cells activated by anti-CD3 and CD28 mAbs; however, 1 and 5 μmol/L baicalein did not induce cell death (Fig. 3A). MTT assays also showed normal cell survival following treatment with 10 μmol/L baicalein (data not shown). Thus, low-dose baicalein (1 and 5 μmol/mL) induced the differentiation of CD4^+^Foxp3^+^ Treg cells without concomitant toxicity.

The induction of CD4^+^Foxp3^+^ T cells by baicalein was verified by real-time RT-PCR analysis of Treg-associated genes (Fig. 3B). Baicalein-treated CD4^+^Foxp3^+^ T cells showed significant up-regulation of granzyme B (*GranB*), *Ctla-4*, *Tgf-β*, and *Foxp3*. These molecules are expressed in/on Treg cells, leading to death of responder T cells through a GranB-dependent mechanism and indirectly blocking T-cell activation via CTLA-4 and TGF-β^26^. Next, we investigated whether baicalein-induced CD4^+^Foxp3^+^ T cells could function as Treg cells by examining the phosphorylation of STATs (STAT 1, 3, 5, and 6). We found that the sham treatment (anti-CD3/-CD28 stimulation) increased the population of phosphorylated STATs, and that baicalein suppressed the activation of STAT-1 (a Th1-related signal), STAT-6 (a Th2-related signal), and STAT-3 (a Th17-related signal) (Fig. 3C). However, phosphorylated STAT-5 (a Treg-related signal) was not decreased in comparison to activated T cells (sham). Thus, baicalein might induce Treg differentiation through STAT-5-independent signaling as phosphorylated STAT-5 was unchanged whereas TGF-β expression was increased in baicalein-induced Treg cells. These results demonstrate that the induction of CD4^+^Foxp3^+^ Tregs by baicalein could suppress the function of other CD4^+^ T cells such as Th1, Th2, and Th17.

We next examined whether baicalein-induced Foxp3^+^CD4^+^ Tregs could suppress the proliferation of effecter Th cells (Teffector). CFSE-labeled CD4^+^CD62L^+^ T cells were co-cultured with Th cells or baicalein-induced Treg cells, which had been pre-cultured with 5 μmol/L baicalein for 3 days, in the presence or absence of TCR stimulation for 3 days. CD4^+^ T cell proliferation was then analyzed by measuring the CFSE signal using FACS. CD4^+^CD62L^+^ T cells co-cultured with baicalein-treated CD4^+^ cells showed significantly reduced proliferation compared to Th cells (59.10% vs. 44.97%), presumably because of their increased Foxp3^+^ Treg population (Supplementary Fig. S1). These results indicated that baicalein possesses activity that might directly generate functional Foxp3^+^CD4^+^ Tregs.

### Baicalein enhances intestinal barrier function in epithelial Caco-2 cells

**Figure 4:**
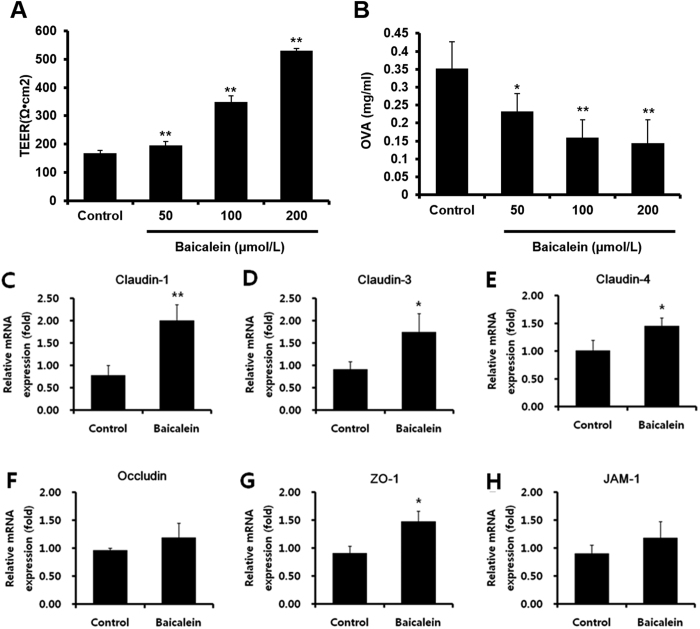
Baicalein enhanced intestinal barrier function in epithelial Caco-2 cells. Caco-2 cell monolayers were incubated with 50, 100, and 200 μmol/L baicalein for 3 h. TEER was measured (**A**), and OVA flux via paracellular diffusion on the basolateral side of the monolayer was detected using ELISA (**B**) mRNA expression levels of TJ-related proteins were analyzed by real-time RT-PCR. Claudin-1 (**C**), claudin-3 (**D**), claudin-4 (**E**), occludin (**F**), ZO-1 (**G**), and JAM-1 (**H**) were evaluated as targets for the modulation of TJ. Each value is presented as means ± SD (*n* = 3). Bars indicate significant difference from the control at *p < 0.05 and **p < 0.01.

To investigate the mechanism of baicalein enhancement of intestinal barrier function, human intestinal epithelial Caco-2 cells were treated with increasing concentrations (50, 100 and 200 μmol/L) of baicalein for 3 h and the TEER value of the cells measured. The TEER values were found to increase in a dose-dependent manner (Fig. 4A). Furthermore, the levels of OVA permeated to the basolateral side of the monolayer via paracellular diffusion were also decreased by baicalein treatment in a dose-dependent manner (Fig. 4B). We next investigated the mechanism by which baicalein regulated TJ-related proteins using the RT^2^ Profiler PCR Array (Qiagen) (Supplementary Information Table S1), and confirmed the expression of TJ-related protein mRNAs in Caco-2 cells using real-time PCR analysis. Baicalein treatment significantly increased the mRNA levels of claudin-1, claudin-3, claudin-4, and ZO-1 (Fig. 4C–H). However, occludin and JAM-1 only showed a non-significant trend toward increased expression following baicalein treatment although these were significantly increased in OVA-induced mice following treatment. These results indicate that baicalein enhances the TJ protein network although the target proteins identified differed slightly *in vitro* and *in vivo*.

### Baicalein induces Treg differentiation via the aryl hydrocarbon receptor

**Figure 5:**
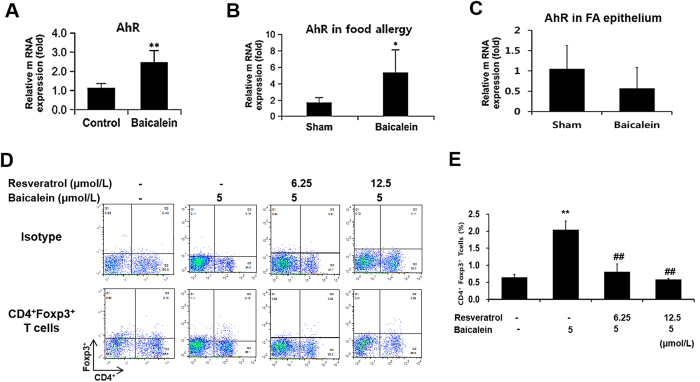
Baicalein induced Treg differentiation via the aryl hydrocarbon receptor. Naive CD4^+^ T cells were cultured in the presence of baicalein (5 μmol/mL), anti-CD3 mAb, and anti-CD28 mAb for 48 h. The mRNA expression of *AhR* in Treg cells induced by baicalein was measured by real-time RT-PCR. In mLN (**B**) and intestinal epithelium (**C**) isolated from the mouse model of food allergy, *AhR* expression was compared between sham and baicalein-treated groups. (**D**) CD4^+^Foxp3^+^ Treg cells were analyzed by flow cytometry after stimulation with TCR and 5 μmol/mL baicalein and resveratrol (an AhR antagonist). Each value represents the means ± SD (*n* = 3). Bars indicate significant difference from the control at *p < 0.05 and **p < 0.01 or from baicalein treatment at ^#^*p* < 0.05 and ^##^*p* < 0.01.

We next investigated how baicalein induces the differentiation of Treg cells from naive CD4^+^ T cells. Baicalein has previously been shown to act as a natural/dietary ligand of the aryl hydrocarbon receptor (AhR) and with agonistic effects^27^. Notably, the mRNA expression of *AhR* was increased in Treg cells induced by baicalein (Fig. 5A). Furthermore, the administration of baicalein up-regulated *AhR* expression in mLNs isolated from food allergy model mice (Fig. 5B). Thus, we explored the AhR dependence of baicalein-induced Foxp3 expression in naive CD4^+^ T cells. Naive CD62L^+^CD4^+^ T cells were cultured with resveratrol (well-known as an AhR antagonist) (Sigma, St. Louis, MO, USA), TCR stimulation, and baicalein. The baicalein-induced CD4^+^Foxp3^+^ Treg population was reduced by competition with resveratrol in a dose-dependent manner (Fig. 5D). These results demonstrated that AhR activation was a requirement for the induction of functional CD4^+^Foxp3^+^ Tregs by baicalein. Furthermore, we confirmed that the enhancement of intestinal barrier function by baicalein was not related to AhR expression and activation in the epithelium from the food allergy mouse model (Fig. 5C).

## Discussion

In this study, we identified that baicalein, a natural flavonoid derived from foods, attenuated food allergic immune responses by inducing Treg cells and enhancing intestinal barrier function. In contrast, we found that baicalin and wogonin, the glycoside and structural analogs of baicalein, respectively were not able to enhance intestinal barrier function or induce CD4^+^Foxp3^+^ Treg cells (Supplemental Information Fig. S2). Therefore, we suggest that baicalein is a functionally distinct compound although baicalin and wogonin represent its glycoside and structural analog, respectively. However, baicalin has been reported to induce Foxp3 expression in a dose-dependent manner (0–40 μmol/L), resulting in Treg differentiation^28^. Here, we found that 40 μmol/L baicalin induced CD4^+^Foxp3^+^ T cells to population levels of 3.89%, but that lower concentrations had no effect on Foxp3 expression. These results indicate that both baicalin and baicalein can induce Treg differentiation although baicalein is the more potent.

As shown in Fig. 3, treatment with 5 μmol/L baicalein led to the generation of CD4^+^Foxp3^+^ T cells without associated cell death whereas some induction of cell death was observed with treatment by 10 μmol/L although the population of Treg cells was maintained. This result was consistent with studies that examined the association between baicalein and apoptosis in T cells^29^. For example, baicalein induced apoptosis in human leukemia HL-60 and Jurkat cells, as confirmed by conversion of MTT (40 μmol/L), release of lactate dehydrogenase (40 μmol/L), and activation of caspase-3 (25 μmol/L)^30^. In this study, we suggest that treatment with 10 μmol/L baicalein is able to induce cell death in mouse CD4^+^ T cells, whereas treatment of <10 μmol/L baicalein can induce an increase in the Treg cell population.

AhR, a well-known receptor against 2, 3, 7, 8-tetrachlorodibenzo-*p*-dioxin, regulates xenobiotic-metabolizing phase 1 and 2 enzymes such as cytochrome P450 3A4 (CYP3A4) and UDP glucuronosyltransferase 1 family polypeptide A1 (UGT1A1)^31^,^32^, and it enhances the expression of p-glycoproteins such as multidrug resistance protein 1 (MDR1) and ATP-binding cassette subfamily B member 1 (ABCB1)^33^. In addition, AhR activation suppresses Th2-type cells through the induction of Th17 or Foxp3^+^ Treg cells although the underlying mechanism is not known^34^,^35^. Baicalein, as a ligand of AhR, also promotes its activation^32^. Therefore, we suggest that the activation of AhR through baicalein binding might induce the differentiation of Treg cells in naive CD4^+^ T cells. Comparatively, curcumin, a dietary AhR compound found in turmeric, attenuates inflammation in asthma and systemic lupus erythematosus by regulating the induction of Treg cells^36^,^37^. In addition, naringenin, a flavonoid in citrus, also induces the differentiation from naive T cells to Treg cells, and was shown to ameliorate the inflammatory symptoms in a DSS (Dextran Sodium Sulfate)-induced colitis model^38^. Therefore, these results together with our findings suggest that the induction of CD4^+^Foxp3^+^ Tregs by baicalein-mediated AhR activation might suppress the function of other CD4^+^ T cells such as Th1, Th2, and Th17. Our results were also consistent with those of previous reports, which reported the efficacy of baicalein in the treatment of atopic dermatitis (Th2-Th1 response), colitis, and rheumatoid arthritis (Th1-Th17 response)^39^,^40^,^41^.

In this study, we also verified that baicalein could enhance the barrier function under various conditions such as the presence of bile salts, IL-4, and IL-6 in Caco-2 cells (Supplementary Fig. S3). Bile salts, IL-4, and IL-6 have been known to lead to intestinal barrier impairment^42^,^43^,^44^. IL-4, which is mainly secreted by T cells, plays a key role in allergic immune responses as a major mediator of allergic disorders. It induces not only the differentiation of CD4^+^ T cells from naive T cells to Th 2 cells, but also the proliferation, differentiation, and activation of B cells, resulting in the production of IgE. Furthermore, IL-4 impairs the intestinal TJ barrier through expression of pore-forming claudin-2 in intestinal T84 cells. Furthermore, IL-6, a pro-inflammatory cytokine, plays a major role in inflammatory and infectious immune responses such as IBD. IL-6 can also lead to intestinal barrier impairment through increasing the TJ protein claudin-2 in intestinal Caco-2 cells. Nevertheless, the treatment of baicalein improved the reduced TEER value in Caco-2 cell monolayers under these conditions. To investigate the mechanism by which baicalein regulated TJ-related proteins, we analyzed TJ-related genes using a PCR array and found that among 84 TJ-related genes, 20 were up-regulated over 2-fold by baicalein, but no gene was down-regulated over 2-fold (Supplementary Information Table S1). In particular, claudin-3 and 4 were increased over 3-fold by baicalein treatment. Claudin-3 and claudin-4 have been known as pore-sealing claudins along with claudin-1, 5, 8, 11, 14, 15, and 19^45^; we also noted increased expression of claudin-3, 4, 11, and 15, although to a lesser degree. The detailed mechanism and physiological meaning of the enhanced expression of these pore-sealing TJ proteins remains to be elucidated in a future study.

It has recently been reported that some polyphenols could enhance intestinal barrier function through the regulation of TJ proteins^46^. In this study, our results showed that baicalein enhanced intestinal barrier function through regulation of TJ proteins as well. In addition, baicalein also induced the differentiation of CD4^+^Foxp3^+^ Treg cells. These results demonstrate that the effects of baicalein for both the enhancement of barrier function and induction of Treg cells can contribute to attenuating food allergic disorder. We suggest that these effects of baicalein might permit the treatment of intestine-related diseases and immune disorders such as allergy, inflammation, and autoimmune disorders. Furthermore, the present study provided evidence for an underlying therapeutic mechanism of baicalein in the treatment for various diseases including food allergy.

In conclusion, we demonstrated that baicalein, a dietary flavonoid, ameliorated OVA-induced food allergic responses by mediating the induction of Treg cells in a mouse model of food allergy. Furthermore, baicalein helped to attenuate the food allergy by regulating TJs in the epithelium. Therefore, the use of baicalein as an anti-allergic agent might contribute to the prevention or/and cure of food allergic disorders and IBD.

## Materials and Methods

### Animals

Female BALB/c mice (*n* = 36), weighing approximately 18 to 20 g, were purchased from Orient Bio (Kyeonggi, Korea). The 4-week-old mice were housed in an air-conditioned room (23 ± 2 °C) with a 12 h light/12 h dark cycle. The animals were allowed free access to food and tap water. All animal experiments were performed in accordance with the Guide for the Experimental Animal Research Laboratory of the Korea Food Research Institute. The protocol was approved by the Korea Food Research Institutional Animal Care and Use Committee (KFRIACUC) on the Ethics of Animal Experiments of the Korea Food Research Institute (Permit Number: KFRI-M-15027). All efforts were made during the animal experiments to minimize suffering.

### Induction of food allergy by oral administration of ovalbumin (OVA)

Mice were divided into naïve (*n* = 5), sham (*n* = 12), baicalein (*n* = 12), and dexamethasone (*n* = 7) groups. To induce an allergic response, mice were sensitized with 20 μg OVA adsorbed in 2 mg/mL Imject Alum (Pierce, Rockford, IL, USA) by intraperitoneal (i.p.) injection on days 0 and 14. Beginning on day 28, mice were orally challenged with 50 mg OVA in saline every 3 days, for five challenges. Baicalein (20 mg/kg) was orally administered daily from day 28 to 40 (Fig. 1A). Diarrhea, anaphylaxis, and rectal temperature were measured as indices of food allergy symptoms as described^47^. Briefly, diarrhea was scored as follows: 0, normal stools; 1, a few wet and unformed stools; 2, a number of wet and unformed stools with moderate perianal staining of the coat; 3, severe and watery stool with severe perianal staining of the coat. Anaphylactic response was also scored as follows: 0, no symptom; 1, reduced activity, trembling of limbs; 2, loss of consciousness, no activity upon prodding; 3, convulsions; 4, death. Rectal temperature was measured using a Thermalert TH5 monitoring thermometer (Physitemp, Clifton, NJ, USA).

### Detection of immunoglobulin E and cytokines by ELISA

To measure IgE and mouse mast cells protease 1 (mMCP-1) levels, serum samples were obtained by collecting blood from the orbital venous plexus. IgE and mMCP-1 levels were measured by ELISA, according to the manufacturer protocol (BD Biosciences, San Jose, CA, USA). The mesenteric lymph nodes (mLN) lymphocytes were assessed for cytokine levels [IFN-γ, IL-12, IL-4, IL-5, IL-13, IL-17, and transforming growth factor beta (TGF-β)] by using a cytokine assay kit (BD Biosciences).

### Histological analysis

Hematoxylin and eosin (H&E) and toluidine blue (TB) staining was performed according to standard procedures (IHC World, Online Information Center for Histochemistry, www.ihcworld.com). All histology was performed by professional pathologist (Dr. Park, Jae-Hak) at the College of Veterinary Medicine, Seoul National University. Briefly, the infiltrated clusters number of inflammatory cells such as neutrophils, eosinophils and mast cell was quantified by some criteria. Scoring is based on the morphological changes of crypt and villi in jejunum and the histopathological changes on inflammation (0 point: normal, 1 point: mild, 2 points: Moderate, 3 points: Severe).

### Real-time reverse transcription-polymerase chain reaction (RT PCR)

mRNA was extracted and purified with the RNeasy Mini Kit (Qiagen, Venlo, the Netherlands), and cDNA was synthesized using the QuantiTect Reverse Transcription Kit (Qiagen). Real time RT-PCR was performed in a Rotor-Gene Q 2plex System (Qiagen) as follows: 10 min at 95 °C, followed by 45 cycles of 30 s at 95 °C, and 30 s at 58–62 °C. Primer sequences are provided in Supplementary Information, Table S2. Transcript levels were normalized to an internal control (GAPDH, HPRT, or β-actin).

### Sorting of naive CD4^+^ T cells and immunofluorescence staining of CD4 and Foxp3

Naive CD4^+^ T cells from the spleens and mLNs of BALB/c mice were isolated using the magnetic-activated cell sorting (MACS) CD4^+^CD62L^+^ T-cell isolation kit (Miltenyi Biotec, Bergisch-Gladbach, Germany), according to manufacturer protocol. The naive CD4^+^ T cells (1 × 10^6^ cells/well) were cultured in 96-well plates in the presence of test reagents. T cell receptor (TCR) stimulation was performed with 10 μg/mL plate-bound anti-CD3 mouse antibody (mAb) (17A2) and 2 μg/mL soluble anti-CD28 mAb (37.51) (BioLegend, San Diego, CA, USA).

CD4 and Foxp3 were stained with anti-CD4-PE and Foxp3 fixation/permeabilization concentrate and diluent (eBioscience, San Diego, CA, USA) according to manufacturer protocol. Data were acquired by flow cytometry on a BD Canto II instrument and analyzed with FlowJo software (Tree Star, Inc., San Carlos, CA, USA).

### Measurement of cell viability using 7-ADD staining

After staining cells for surface CD4 molecules, the cells were washed twice with FACS buffer. The cells resuspended with 100 μL of FACS buffer were incubated with 5 μL of 7-AAD staining solution (eBioscience, San Diego, CA, USA) for 15 min RT before analyzing cells on a flow cytometer.

### Western blotting

Naive CD4^+^ T cells (1 × 10^7^ cells/well) were cultured with 5 μmol/L baicalein in 12-well plates in the presence 10 μg/mL plate-bound anti-CD3 mAb and 2 μg/mL soluble anti-CD28 mAb for 48 h. The cells were lysed with RIPA buffer including protease inhibitor cocktail (Bio-Rad, Richmond, CA, USA). Quantified proteins (20 μg) were separated by sodium dodecyl sulphate-polyacrylamide gel electrophoresis and transferred to polyvinylidene difluoride membranes. After blocking with 5% skim milk, the membranes were incubated with specific primary [total- and phospho-STAT1 (1:1000), STAT3 (1:2000), STAT5 (1:1000), and STAT6 (1:1000)] (Cell Signaling Technology, Beverly, MA, USA) and secondary antibodies (1:3000) (Bio-Rad). The membranes were subjected to an enhanced chemiluminescence reaction and signals were detected using an AE9300 instrument (ATTO, Tokyo, Japan).

### Cell culture and evaluation of intestinal barrier function

Caco-2 cell line (ATCC HTB 37) was obtained from the American Type Culture Collection (Rockville, MD, USA) and was cultured at 37 °C in humidified air containing 5% CO_2_. The cells were seeded at a density of 2 × 10^5^ cells/mL on a 12-transwell or 24-well plate (Costar, Corning, NY, USA) and allowed to grow for 3 weeks prior to the experiments.

The integrity of the resultant Caco-2 cell monolayers was checked by measuring the transepithelial electrical resistance (TEER) using a Millicell-ERS device (Millipore, Bedford, MA, USA). The monolayers were used when their TEER values were between 300–500 Ω cm^−2^. The TEER value and amount of OVA influx were detected and used as measures for the evaluation of intestinal barrier function as previously described^48^.

### Statistical analysis

Data are presented as the means ± standard deviation (SD) of triplicate measurements. Differences between experimental data were analyzed by ANOVA followed by an F-protected Fisher’s least significant difference test. *p*-values < 0.05 were considered statistically significant.

## Additional Information

**How to cite this article**: Bae, M.-J. *et al.* Baicalein induces CD4^+^Foxp3^+^ T cells and enhances intestinal barrier function in a mouse model of food allergy. *Sci. Rep.* **6**, 32225; doi: 10.1038/srep32225 (2016).

## Supplementary Material

Supplementary Figure 1

Supplementary Figure 2

Supplementary Figure 3

Supplementary Table 1

Supplementary Table 2

## Figures and Tables

**Table 1:** Baicalein enhanced intestinal barrier function in OVA-induced mice.

Gene	Tight Junction Transcript Expression			
	Group			
	Naive	Sham	Baicalein	Dexamethasone
Occludin	1.06 ± 0.59	0.64 ± 0.57	3.55 ± 2.47**	0.58 ± 0.32
Claudin-1	1.00 ± 0.52	0.55 ± 0.22	1.05 ± 0.54*	0.36 ± 0.18
ZO-1	1.05 ± 0.44	0.82 ± 0.25	1.10 ± 0.23*	0.68 ± 0.18
JAM-1	1.03 ± 0.22	0.86 ± 0.18	1.08 ± 0.25*	1.01 ± 0.28

Each value represents the means ± SD (*n* = 5–12). *p < 0.05 and **p < 0.01 vs. Sham. OVA, ovalbumin.
