# Supplementary material for: Baicalein induces CD4+Foxp3+ T cells and enhances intestinal barrier function in a mouse model of food allergy
Source: Sci Rep. 2016 Aug 26;6:32225. doi: 10.1038/srep32225 (PMC4999817; doi:10.1038/srep32225)
Supplement: Supplementary Figure 1 [file srep32225-s1.docx]

**Baicalein induces CD4^+^Foxp3^+^ T cells and enhances intestinal barrier function in a mouse model of food allergy**

Min-Jung Bae, Hee Soon Shin, Hye-Jeong See, Sun Young Jung, Da-Ae Kwon, Dong-Hwa Shon

**Supplementary Figure 1**

**
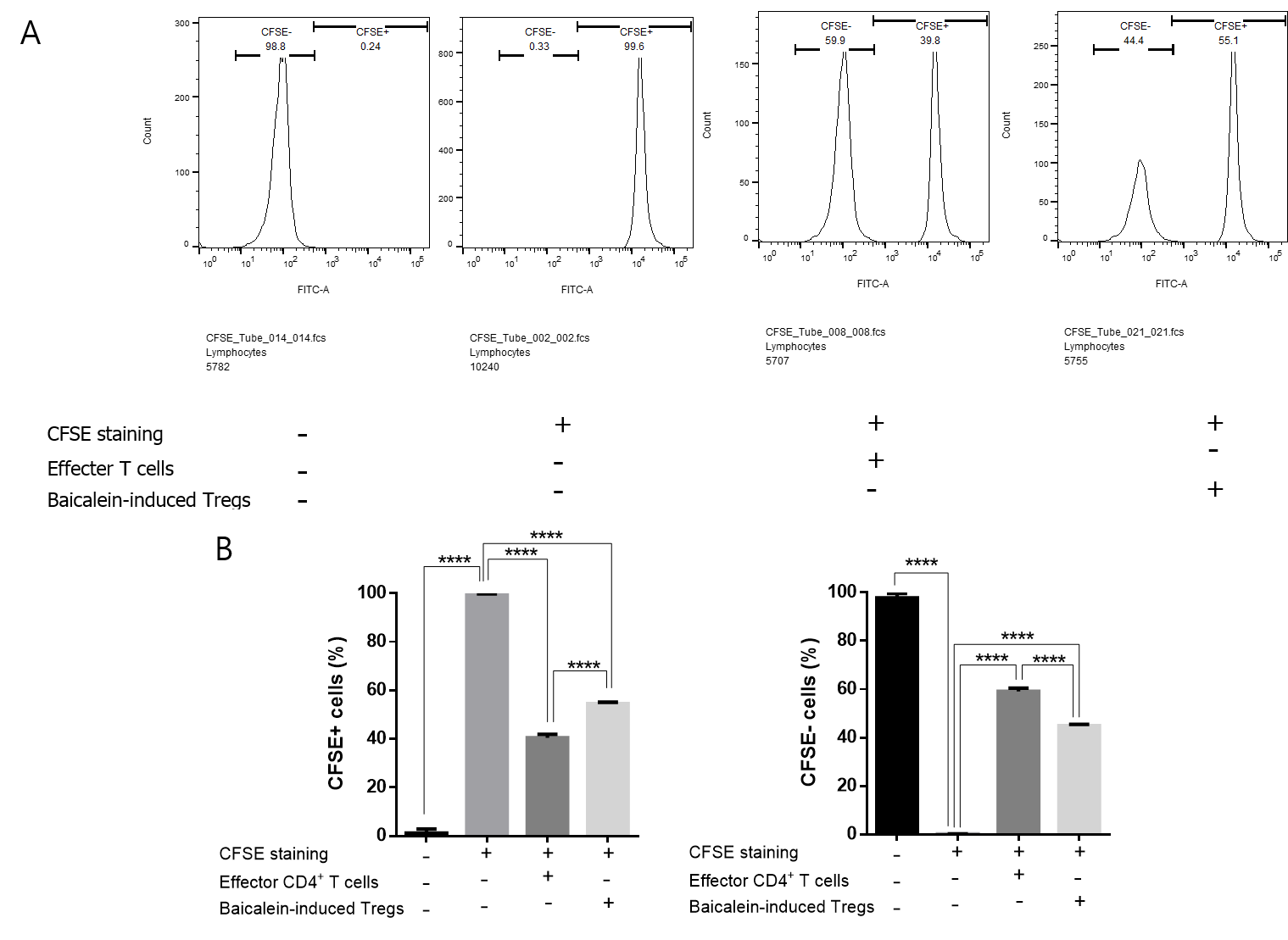
**

**Supplementary Figure 1. Baicalein affects the generation of functional Foxp3^+^ T regulatory cells.**

**A.** CD4^+^ T cells cultured in the presence of 0−5 μmol/L baicalein for 3 days were co-cultured with CD4^+^CD62L^+^ naive T cells labeled with CFSE (5 µM), 2 μg/mL plate-bound anti-CD3 mAb, and 2 μg/mL soluble anti-CD28 mAb. The CFSE^+^ population was then analyzed using FACS. A plot from one representative experiment shows the frequency of CFSE^+^CD4^+^ T cells. **B and C.** Data are presented as the mean ± SD of triplicate determinations of CFSE^+^CD4^+^ T cells and CFSE^-^CD4^+^ T cells, and were analyzed by one way analysis of variance (ANOVA) followed by the Dunnett’s post-hoc test.: *P < 0.05, **P < 0.005, ***P < 0.001 and ****P < 0.0001 vs. control, respectively.
