# Supplementary material for: Baicalein induces CD4+Foxp3+ T cells and enhances intestinal barrier function in a mouse model of food allergy
Source: Sci Rep. 2016 Aug 26;6:32225. doi: 10.1038/srep32225 (PMC4999817; doi:10.1038/srep32225)
Supplement: Supplementary Figure 2 [file srep32225-s2.docx]

**Baicalein induces CD4^+^Foxp3^+^ T cells and enhances intestinal barrier function in a mouse model of food allergy**

Min-Jung Bae, Hee Soon Shin, Hye-Jeong See, Sun Young Jung, Da-Ae Kwon, Dong-Hwa Shon

**Supplementary Figure 2**

**
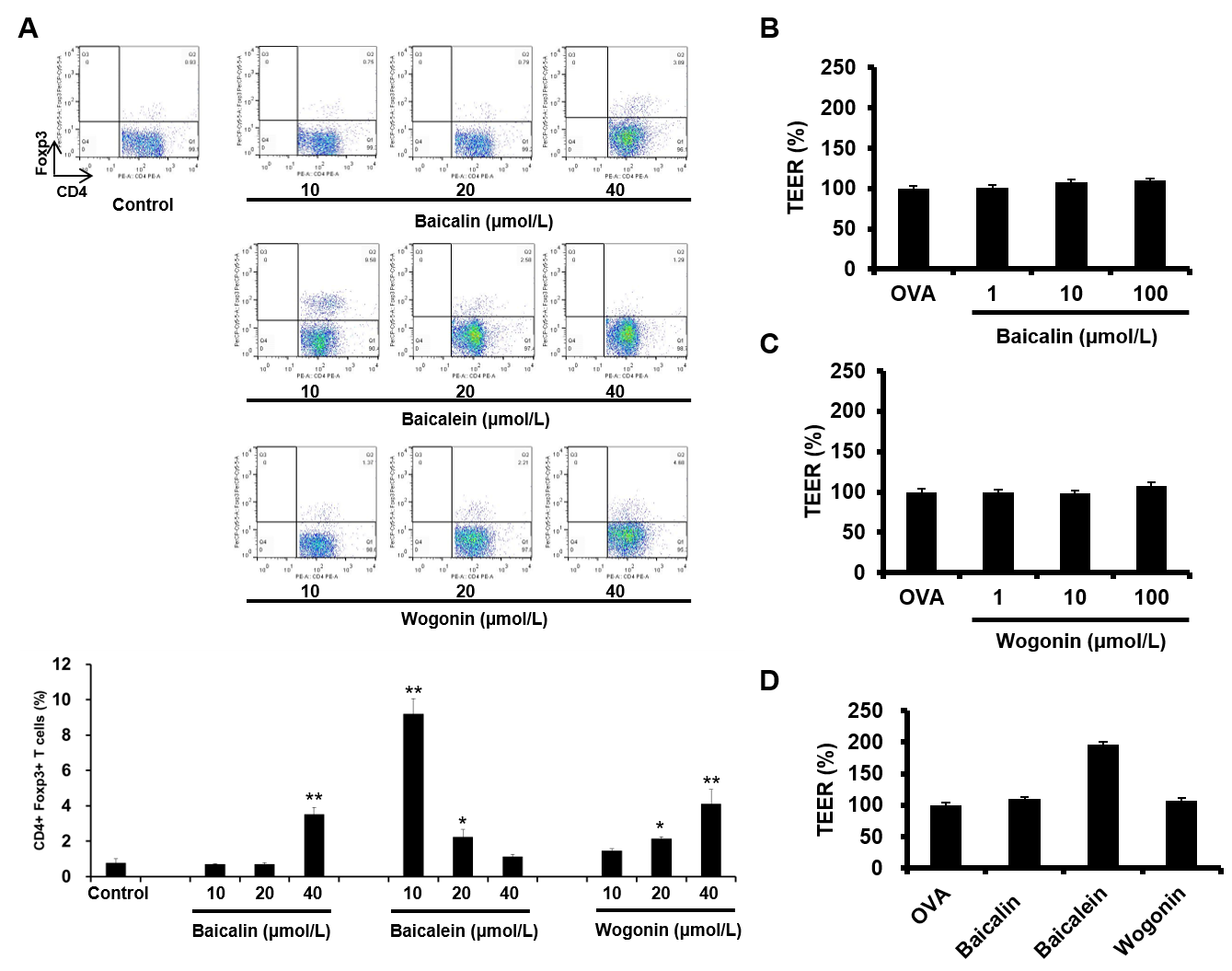
**

**Supplementary Figure 2**

**Effects of baicalein and its analogs (baicalein and wogonin) on induction of CD4^+^Foxp3^+^ Treg cells and enhancement of intestinal barrier function**

**A.** Naïve CD4^+^ T cells (CD4^+^CD62L^+^ T cells) from the spleen and mLN were treated with 0–40 µmol/L baicalin, baicalein, or wogonin for 72 h, stained for CD4 and Foxp3, and analyzed by flow cytometry. **B-D.** The Caco-2 cell monolayers were incubated with 1, 10, and 100 µmol/L baicalin, baicalein, or wogonin for 3 h. Intestinal barrier function was evaluated by measuring TEER values. Each value is presented as mean ± SD (*n* = 3). Bars are significantly different from the control at **P <* 0*.*05 and ***P <* 0*.*01.
