# Supplementary material for: Baicalein induces CD4+Foxp3+ T cells and enhances intestinal barrier function in a mouse model of food allergy
Source: Sci Rep. 2016 Aug 26;6:32225. doi: 10.1038/srep32225 (PMC4999817; doi:10.1038/srep32225)
Supplement: Supplementary Figure 3 [file srep32225-s3.docx]

**Baicalein induces CD4^+^Foxp3^+^ T cells and enhances intestinal barrier function in a mouse model of food allergy**

Min-Jung Bae, Hee Soon Shin, Hye-Jeong See, Sun Young Jung, Da-Ae Kwon, Dong-Hwa Shon

**Supplementary Figure 3**

**
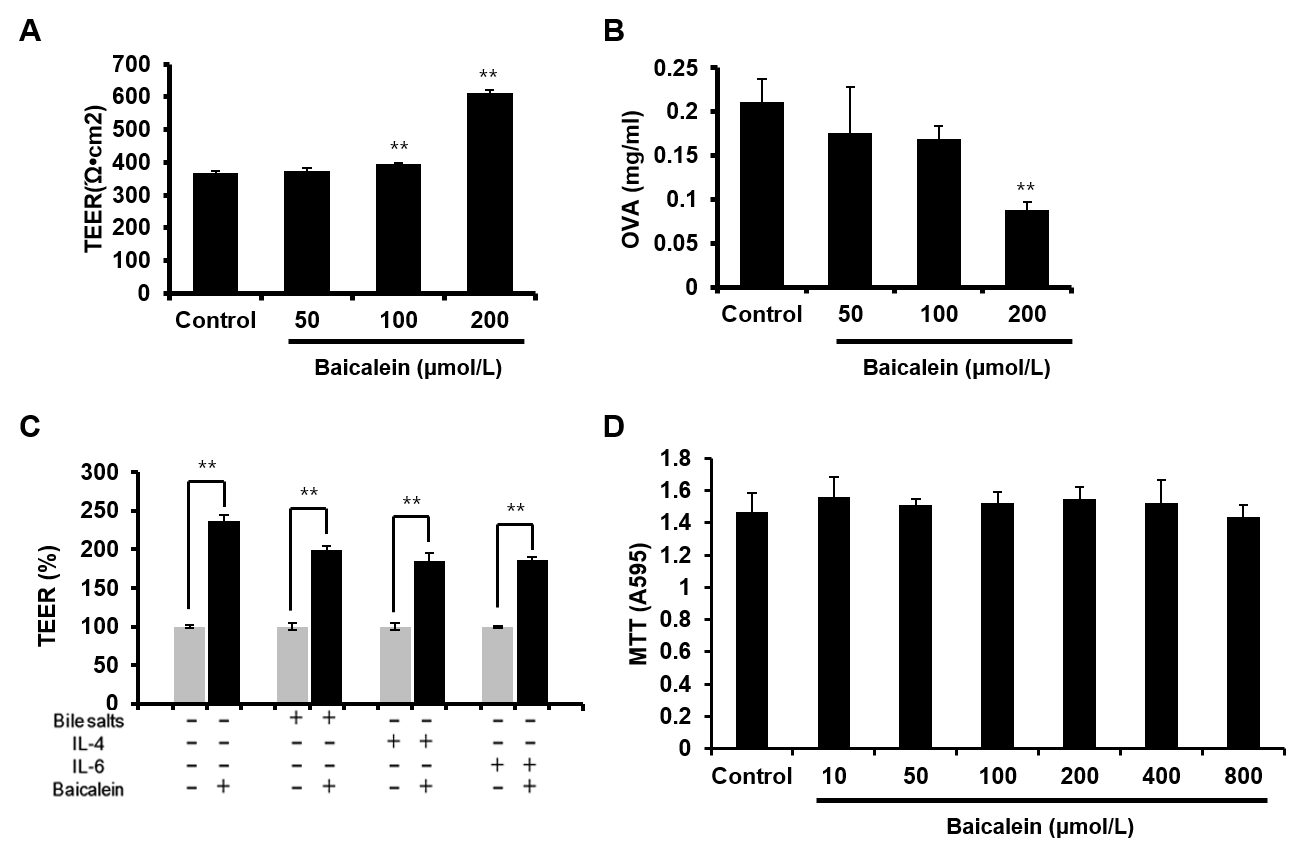
**

**Supplementary Figure 3**

**Enhancing effects of baicalein on intestinal barrier functions in various conditions**

**A**. The Caco-2 cell monolayer were incubated with 50, 100, and 100 µmol/L baicalein in absence of bile salts for 3 h. Intestinal barrier function was evaluated by measuring TEER values. **B**. OVA flux on the basolateral side of the monolayer via paracellular diffusion was detected using ELISA. **C**. The Caco-2 cell monolayer were incubated with baicalein in presence or absence of bile salts, IL-4, and IL-6 for 3 h. **D**. Cytotoxicity by baicalein treatment was measured by MTT assay. Each value is presented as mean ± SD (*n* = 3). Bars are significantly different from the control at ***P <* 0*.*01.
