# Supplementary material for: Baicalein induces CD4+Foxp3+ T cells and enhances intestinal barrier function in a mouse model of food allergy
Source: Sci Rep. 2016 Aug 26;6:32225. doi: 10.1038/srep32225 (PMC4999817; doi:10.1038/srep32225)
Supplement: Supplementary Table 1 [file srep32225-s4.docx]

**Baicalein induces CD4+Foxp3+ T cells and enhances intestinal barrier function in a mouse model of food allergy**

Min-Jung Bae, Hee Soon Shin, Hye-Jeong See, Sun Young Jung, Da-Ae Kwon, Dong-Hwa Shon

**Supplementary Table 1**

**mRNA expressions of TJ-related protein by PCR array ( >2folds)**

| ***Gene*** | ***Genebank Number*** | ***Fold Change*** |
| --- | --- | --- |
| CLDN3 | **NM_001306** | **3.249** |
| ACTN3 | **NM_001104** | **3.095** |
| CDLN4 | **NM_001305** | **3.074** |
| CLDN11 | **NM_005602** | **2.969** |
| F11R | **NM_016946** | **2.657** |
| CTNNB1 | **NM_001904** | **2.567** |
| MARK2 | **NM_004954** | **2.514** |
| CLDN15 | **NM_014343** | **2.395** |
| MLLT4 | **NM_001040000** | **2.266** |
| TJAP1 | **NM_080604** | **2.174** |
| ACTN2 | **NM_001103** | **2.174** |
| ASH1L  CGN  ACTN4  ARHGEF2  LLGL1  TJP2  SYMPK  CLDN9   | **NM_018489**  **NM_020770**  **NM_004924**  **NM_04723**  **NM_004140**  **NM_004817**  **NM_004819**  **NM_020982**  **NM_005231** | **2.159**  **2.114**  **2.085**  **2.085**  **2.085**  **2.085**  **2.071**  **2.042**  **2.0** |
