# Supplementary material for: Baicalein induces CD4+Foxp3+ T cells and enhances intestinal barrier function in a mouse model of food allergy
Source: Sci Rep. 2016 Aug 26;6:32225. doi: 10.1038/srep32225 (PMC4999817; doi:10.1038/srep32225)
Supplement: Supplementary Table 2 [file srep32225-s5.docx]

**Baicalein induces CD4^+^Foxp3^+^ T cells and enhances intestinal barrier function in a mouse model of food allergy**

Min-Jung Bae, Hee Soon Shin, Hye-Jeong See, Sun Young Jung, Da-Ae Kwon, Dong-Hwa Shon

**Supplementary Table 2**

**Primer sequences used for real-time RT-PCR.**

| ***Primer sequences*** | ***Forward (5'–3')*** | ***Reverse (5'–3')*** |
| --- | --- | --- |
| Granzyme B | **CTCCTAAAGCTGAAGAGTAAGG** | **TTTAAAGTAGGACTCACACTCCC** |
| CTLA-4 | **AGAACCATGCCCGGATTCTG** | **CATCTTGCTCAAAGAAACAGCAG** |
| Foxp3 | **CAGCTGCCTACAGTGCCCCTAG** | **CATTTGCCAGCAGTGGGTAG** |
| AhR | **TGCACAAGGAGTGGACGA** | **TGCACAAGGAGTGGACGA** |
| IL-4 | **CGAAGAACACCACAGAGAGTGAGCT** | **GACTCATTCATGGTGCAGCTTATCG** |
| IFN-γ | **AGCGGCTGACTGAACTCAGATTGTAG** | **GTCGCTTCGTTGATCACAA** |
| IL-17 | **TTCATCTGTGTCTCTGATGCT** | **TTGACCTTCACATTCTGGAG** |
| GATA-3 | **GAAGGCATCCAGACCCGAAAC** | **GACTCATTCATGGTGCAGCTTATCG** |
| T-bet | **TGCCTGCAGTGCTTCTAACA** | **TGCCCCGCTTCCTCTCCAACCAA** |
| ROR-γt | **CCGCTGAGAGGGCTTCAC** | **TGCAGGAGTAGGCCACATTACA** |
| GAPDH | **TGAACGGGAAGCTCACTGG** | **TCCACCACCCTGTTGGTGTA** |
| HPRT  Occludin  Claudin-1  Claudin-3  Claudin-4  ZO-1  JAM-1  β-actin | **TTATGGACAGGACTGAAAGAC**  **GCTGTGATGTGTGTTGACTG**  **TCTACGAGGGACTGTGGATG**  **AAGCCGAATGGACAAAGAA**  **CGCTACTCTTGCCATTACG**  **AGGACACCAAAGCATGTGAG**  **ACCCTCCCTCCTTTCCTTAC**  **TCATGAAGTGTGACGTGGACATC** | **GCTTTAATGTAATCCAGCAGGT**  **CAAGGAGGTCCATCTGGCAG**  **GCTGAGGAACGACTTAGACT**  **GTGACGTCGATGAACGGTC**  **GTTCAGTACCACACGACTCA**  **ACATTGGTCGTCCTTACGG**  **CCTAACCCGTTCTCAGGATC**  **TGCATCCTGTCGGCAATG** |
